# Allogeneic Hematopoietic Stem Cell Transplantation for a *BCR-FGFR1* Myeloproliferative Neoplasm Presenting as Acute Lymphoblastic Leukemia

**DOI:** 10.1155/2012/620967

**Published:** 2012-10-02

**Authors:** Karl Haslam, Stephen E. Langabeer, Johanna Kelly, Natasha Coen, Niamh M. O'Connell, Eibhlin Conneally

**Affiliations:** ^1^Cancer Molecular Diagnostics, Central Pathology Laboratory, St. James's Hospital, Dublin 8, Ireland; ^2^National Centre for Medical Genetics, Our Lady's Children's Hospital, Dublin 12, Ireland; ^3^Department of Haematology, St. James's Hospital, Dublin 8, Ireland

## Abstract

Hematopoietic myeloproliferative neoplasms (MPNS) with rearrangements of the receptor tyrosine kinase *FGFR1* gene, located on chromosome 8p11, are uncommon and associated with diverse presentations such as atypical chronic myeloid leukemia, acute myeloid leukemia, or an acute T- or B-lymphoblastic leukemia, reflecting the hematopoietic stem cell origin of the disease. A review of MPN patients with the t(8;22) translocation that results in a chimeric *BCR-FGFR1* fusion gene reveals that this disease either presents or rapidly transforms into an acute leukemia that is generally unresponsive to currently available chemotherapeutic regimens including tyrosine kinase inhibitors (TKIS). The first case of a rare *BCR-FGFR1* MPN presenting in a B-acute lymphoblastic phase who underwent allogeneic hematopoietic stem cell transplantation (HSCT) with a subsequent sustained complete molecular remission is described. Allogeneic HSCT is currently the only available therapy capable of achieving long-term remission in *BCR-FGFR1* MPN patients.

## 1. Introduction

The fibroblast growth factor receptor 1 (*FGFR1*) gene, located on chromosome 8p11, encodes a receptor tyrosine kinase. Upon ligand binding, receptor dimerization occurs with subsequent autophosphorylation and recruitment of target proteins PLC-*γ* and effectors of the RAS/MAPK signalling pathway essential for normal cellular proliferation and differentiation [1]. Rearrangements of *FGFR1* are associated with a heterogeneous set of hematological malignancies thought to be derived from a pluripotent haematopoietic stem cell that may present as an atypical myeloproliferative neoplasm, a lymphoblastic lymphoma, or in transformation to either a myeloid or lymphoid acute leukemia in which eosinophilia is characteristic but not invariable [2]. The defining molecular feature of this group of neoplasms is evidence of balanced translocations of *FGFR1* to one of several genes, most frequently *ZMYM2* at 13q11, and also to *FOP* (6q27), *CEP110* (9q33), *BCR* (22q11), and several other partner genes [2]. The resulting fusion genes retain the C-terminal portion of *FGFR1* and lead to constitutive ligand-independent aberrant tyrosine kinase activation with some evidence that the partner gene of *FGFR1* influences the phenotype of the disease [3]. The disease is associated with a high risk of progression to acute leukemia and in the absence of an available specific TKI therapy, the only potentially curative option is allogeneic HSCT which should be considered even in those eligible patients in the chronic phase of the disease [4, 5].

The recurrent t(8; 22)(p11; q11) translocation, that results in a *BCR-FGFR1* fusion, has to date been reported in only twelve adult patients [6–16] and one pediatric patient [17] (Table 1). Therapy has varied depending on the phenotype of the disorder with patients presenting in an acute leukemic phase rarely achieving long-term remission. We present a case of *BCR-FGFR1* MPN which presented in an acute lymphoblastic phase demonstrating a favorable outcome following allogeneic HSCT.

## 2. Case Report

**Figure 1:**
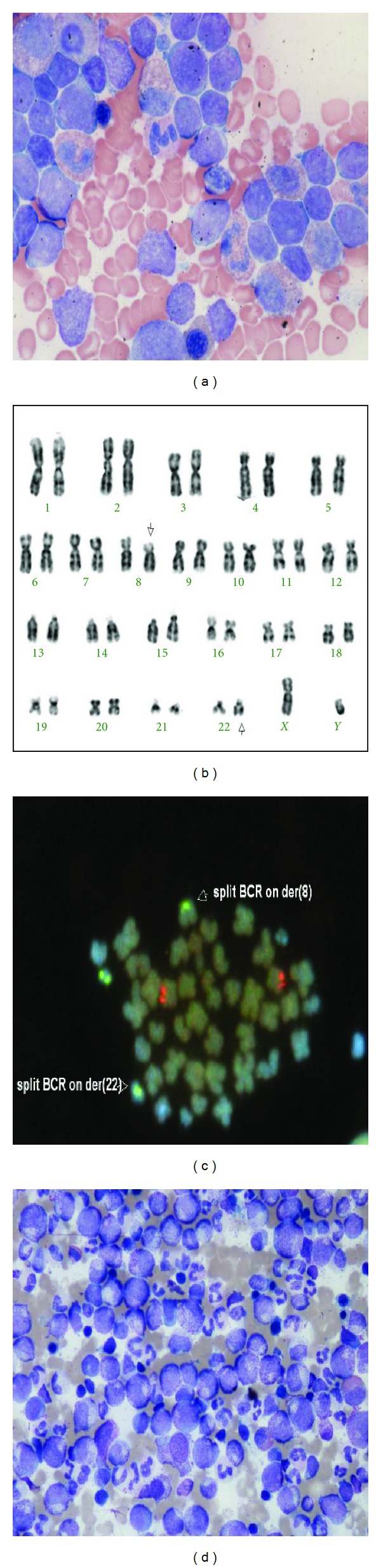
(a) Presenting bone marrow aspirate showing large lymphoblasts; (b) karyotype depicting t(8; 22) translocation highlighted by arrows; (c) FISH demonstrating a split *BCR* signal on chromosomes 8 and 22; (d) bone marrow aspirate with myeloid hyperplasia consistent with an atypical MPN.

**Figure 2:**
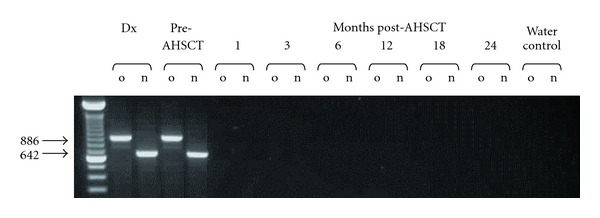
RT-PCR of *BCR-FGFR1* transcripts at diagnosis and throughout treatment course. Dx: diagnosis; o: outer; n: nested.

A 21-year-old male presented with a facial palsy and peripheral leukocytosis. Physical examination revealed features of meningism but no other abnormalities. His presenting FBC revealed hemoglobin 13.1 g/L, white cell count 78.2 × 10^9^/L, and platelets of 167 × 10^9^/L. A review of the peripheral blood film showed predominantly neutrophils, with an increase in basophils, metamyelocytes, and myelocytes and occasional circulating blast cells. His LDH at presentation was elevated at 971 IU/L (normal range 230–450 IU/L). Bone marrow aspirate examination showed a hypercellular marrow with an infiltrate of lymphoid appearing blast cells (62%) (Figure 1(a)). Multicolor flow cytometry of these blast cells demonstrated expression of CD34, HLA-DR, CD10, CD19, CD71, nuclear TdT and cytoplasmic CD79a and CD22, consistent with precursor B-cell acute lymphoblastic leukemia (B-ALL). Flow cytometry also demonstrated a population of dual CD19/CD34 positive cells in cerebro-spinal fluid, corroborating central nervous system involvement. The bone marrow karyotype showed a balanced translocation between chromosomes 8 and 22 as the primary karyotypic event with evidence of clonal evolution in the form of a further whole arm translocation, a dicentric and a ring chromosome in eleven metaphases analysed: 46,XY,t(8; 22)(p12; q11)[8]/45,idem,der(3; 9)(q10; q10),dic(7; 11)(p11; q13),+r [cp3] (Figure 1(b)). Fluorescent *in situ* hybridization (FISH) analysis demonstrated the presence of a split *BCR* (22q11) signal, with signals located on both the der(8) and der(22) in 170/200 cells (Figure 1(c)). Additional FISH with an 11q23 probe set showed loss of an *MLL* signal in a smaller subset of cells (59/200). Seminested reverse transcriptase-PCR using forward primers BCR-e1-A and BCR-E1+ and reverse primer FGFR9- [6, 18] confirmed the presence of *BCR-FGFR1* transcripts at the molecular level without expression of the reciprocal *FGFR1-BCR* fusion (Figure 2).

The patient was initially treated according to an adolescent ALL protocol (UKALL 2003) [19]. After one cycle of chemotherapy, the bone marrow was hypercellular marrow consistent with a myeloproliferative disorder with resolution of the ALL. Interphase FISH demonstrated persistence of the t(8; 22) clone in 262/300 cells. In the absence of a human leukocyte antigen compatible sibling, a search for an unrelated donor was initiated. The patient continued on the UKALL 2003 protocol and received phase II induction and high-dose methotrexate. Four months after diagnosis, prior to the planned allogeneic HSCT, the patient developed a rapidly increasing peripheral white cell count with a review of the blood film consistent with an accelerated phase of the myeloproliferative component of his disease. The bone marrow aspirate showed evidence of the primary MPN with marked myeloid hyperplasia, increased numbers of megakaryocytes, and an absence of blasts (Figure 1(d)). Cytogenetics confirmed the presence of t(8; 22) as the sole aberration in all 30 cells analysed. The patient received FLAG-Ida chemotherapy which reduced the cellularity and rapidly progressed to a myeloablative, mismatched, unrelated donor HSCT. His conditioning regimen consisted of cyclophosphamide/total body irradiation with antithymocyte globulin added due to the B-antigen mismatch. The initial transplant course was unremarkable; however, on engraftment, he developed grade II skin graft-versus-host disease (GVHD) and required therapy with corticosteroids. *BCR-FGFR1* RT-PCR was performed at regular intervals and demonstrated molecular remission at one month after allogeneic HSCT with concurrent peripheral blood full-donor chimerism [20]. The patient remains clinically well two years after allogeneic HSCT with extensive chronic GVHD requiring ongoing therapy with Tacrolimus, low-dose corticosteroids, and mycophenolate mofetil with sustained undetectable *BCR-FGFR1* transcripts (Figure 2). Despite this, the patient has returned to full-time education and his current Karnofsky score is 80%.

## 3. Discussion

*BCR-FGFR1* MPN is a rare disease that manifests as diverse phenotypes attesting to the proposal that the transforming genetic defect occurs in a pluripotent hematopoietic stem cell capable of multilineage differentiation and further corroborated in the patient described herein whereby initial successful treatment of the presenting acute leukemia revealed the underlying MPN. A recently described murine model recapitulates the human disease with bilineage myeloid and B-cell involvement [21]. Though usually presenting as an atypical chronic myeloid leukemia, the present case is the fourth *BCR-FGFR1*-positive patient diagnosed with lymphoblastic leukemia (Table 1) suggesting that specific domains or amino acid residues of the translocation partner protein of FGFR1 may not be the dominant factor that influences the presenting phenotype [22]. Cellular mechanisms responsible for this phenotypic diversity may include the absolute stage of stem cell differentiation in which the genetic lesion arises, the intensity of resulting kinase activation or perturbations in pathways analogous to those known to be involved in BCR-ABL1-dependent blast crisis transformation of CML such as a block of myeloid differentiation, enhanced self-renewal, or proliferation and survival characteristics of leukemic stem cells [3, 23].

In the postallogeneic HSCT period, molecular monitoring utilizing both *BCR-FGFR1* RT-PCR and microsatellite donor chimerism analysis was performed to detect any evidence of minimal residual disease (MRD). Donor lymphocyte infusions have been shown to offer potential in eliminating MRD by mediating graft-versus-leukemia activity in a previous case of *ZMYM2-FGFR1* MPN [5] and which may have been achieved in this case by the ongoing chronic GVHD.

Several tyrosine kinase inhibitors have been shown to be active *in vitro* against transformed cell lines and primary cells bearing *FGFR1* kinase fusions [24, 25] but have limited long-term efficacy *in vivo* [26, 27]. Development of more potent TKIs, such as the broad-spectrum ponatinib, active against resistant mutations of *FLT3* and *BCR-ABL1* in acute and chronic myeloid leukemia, respectively, may be of future clinical benefit in *FGFR1* MPN [28–30]. 

This is the first description of a patient with a *BCR-FGFR1* MPN presenting with B-ALL who has undergone allogeneic HSCT: three previously described patients presenting with either an atypical MPN or a T-lymphoblastic lymphoma all achieved a complete remission and were alive at last reported followup after allogeneic HSCT. This case confirms the stem cell nature of t(8; 22) MPN and provides further evidence that, in the absence of currently available efficacious TKI therapy, allogeneic HSCT is the only potentially curative option for this clinically aggressive disease.

## Figures and Tables

**Table 1:** Summary of presenting and clinical features of previously reported *BCR-FGFR1* patients.

Case	Reference	Age	Gender	Diagnosis	Treatment	Response	Outcome
(1)	[6]	65	F	Atypical CML	HU/IFN	Unknown	Unknown
(2)	[6]	51	F	Atypical CML	HU/IFN	Unknown	Unknown
(3)	[7]	75	M	Atypical CML			
				AML transformation	HU/cytarabine	Partial hematological response	Unknown
(4)	[8]	74	F	Atypical CML	HU	Partial hematological response	Unknown
(5)	[9]	68	M	Atypical MPN			
				LBC	HU	No hematological response	Unknown
(6)	[10]	58	F	Atypical CML			
				AML transformation		Unknown	Unknown
				Atypical CML	HU/IFN	Minimal hematological and cytogenetic response	
(7)	[11]	56	F		Cytarabine/IFN/ATO	No hematological response	
				AML transformation	Daunorubicin/cytarabine	Bone marrow aplasia	Deceased
(8)	[12]	50	F	CML AP→BC	Unknown	Unknown	Deceased
(9)	[13]	70	F	B-ALL	Induction/consolidation/ maintenance chemotherapy	Morphological remission yet persistent t(8;22)	
				Atypical MPN	HU/maintenance	Disease progression	Deceased
(10)	[14]	57	F	Atypical MPN	AHSCT	Remission for 42 months post AHSCT	Alive
(11)	[15]	59	M	T-LBL/MPN	Cytarabine/daunorubicin/ vincristine/prednisone	Persistent t(8;22)	
					AHSCT	Cytogenetic remission	Alive
				B-ALL	HyperCVAD	Remission yet leukocytosis and splenomegaly	
(12)	[16]	43	M	Atypical CML	HU/Sorafenib	Partial haematological response/relapse	
				B-ALL	Sorafenib/FLA-Ida (no G-CSF)	Persistent B-ALL/AML transformation	Deceased
(13)	[17]	8	M	MDS/MPN	HU/AHSCT	Remission for 54 months post AHSCT	Alive

CML: chronic myeloid leukemia; HU: hydroxyurea; IFN: interferon-alpha; AML: acute myeloid leukemia; MPN: myeloproliferative neoplasm; LBC: lymphoid blast crisis; ATO: arsenic trioxide; AP: accelerated phase; BC: blast crisis; ALL: acute lymphoblastic leukemia; T-LBL, T-lymphoblastic lymphoma; MDS: myelodysplastic syndrome; AHSCT: allogeneic hematopoietic stem cell transplantation; hyperCVAD: hyper-fractionated cyclophosphamide, vincristine, doxorubicin, dexamethasone; FLA-Ida: fludarabine, cytarabine, idarubicin.
